# Imaging mimickers of cholangiocarcinoma in the extra-hepatic biliary tree: a pictorial review with diagnostic clues

**DOI:** 10.1007/s00261-026-05612-z

**Published:** 2026-06-23

**Authors:** Laís de Brito Oliveira, Amanda Bastos Guimarães, Luana Lemos Alves, Igor J. N. Leite, Luiz Alberto Coutinho de Freitas Lima, Viviane Brandão Amorim, Daniella Braz Parente

**Affiliations:** 1 Radiology Department, Instituto Nacional do Câncer, Rio de Janeiro, Brazil; 2 Radiology Department, Fleury S.A. (Brazil), Rio de Janeiro, Brazil; 3 Radiology Department, Federal University of Rio de Janeiro, Rio de Janeiro, Brazil

**Keywords:** Common bile duct, Cholangiocarcinoma, MRCP, Biliary stricture, Biliary obstruction, Mimics, Differential diagnosis, MRI, CT

## Abstract

Distal and perihilar biliary strictures and intraductal lesions frequently trigger immediate concern for cholangiocarcinoma (CCA). However, a broad spectrum of benign, inflammatory, infectious, vascular, and extrinsic processes can produce overlapping imaging appearances. Typical CCA of the common bile duct (CBD) commonly manifests as a long, irregular, asymmetric stricture with conspicuous mural thickening and progressive enhancement. These lesions may be accompanied by periductal soft tissue and vascular encasement. In contrast, mimickers more often demonstrate short, smooth, symmetric narrowing; obstructive causes such as stones or periampullary diverticula; extrinsic compression such as lymphadenopathy or portal cavernoma; inflammatory patterns with interval improvement; congenital ductal ectasia; or characteristic systemic/infectious findings. Careful pattern recognition on MRCP and contrast-enhanced MRI and CT integrating stricture morphology, enhancement behavior, vascular relationships, and temporal evolution provide a reliable framework to distinguish malignant CBD cholangiocarcinoma from benign mimics. This review presents a structured approach to differentiate CBD cholangiocarcinoma from key mimickers using MRI/MRCP and CT.

## Introduction

CCA of the extrahepatic biliary tree is an uncommon yet aggressive malignancy [1]. Its development is linked to chronic inflammation and repeated epithelial injury, which may lead to dysplasia and malignant transformation, often in the setting of established risk factors, such as chronic liver disease, primary sclerosing cholangitis, inflammatory bowel disease, parasitic infections, hepatolithiasis, and other chronic biliary inflammatory conditions [2]. Additional risk factors include choledochal cysts, other congenital or acquired biliary tract anomalies, exposure to industrial toxins, and hereditary cancer syndromes [3].

When imaging reveals bile duct dilatation or an intraductal abnormality, particularly in the distal and perihilar ducts, where malignant strictures are common, CCA is suspected [1]. Early diagnosis is essential to guide therapy and avoid unnecessary or inappropriate procedures [4]. However, differentiating malignant from benign biliary strictures remains a major diagnostic challenge. Numerous benign, inflammatory, infectious, and extrinsic processes may mimic cholangiocarcinoma, especially when presenting as ductal wall thickening or luminal narrowing without a discrete mass [5–7].

This review presents a practical imaging framework and a case-based gallery of CBD cholangiocarcinoma mimickers highlighting how careful analysis of imaging features can help distinguish malignant CBD cholangiocarcinoma from its key mimickers and support confident radiologic decision-making. While some mimickers, such as choledocholithiasis in typical presentations are clinically straightforward, others pose substantial diagnostic challenges: PSC dominant strictures, IgG4-related sclerosing cholangitis, post-surgical fibrotic strictures, and portal cavernoma cholangiopathy remain among the most difficult entities to reliably distinguish from cholangiocarcinoma on imaging alone, and receive particular attention in this review.

## Normal anatomy and an imaging-first approach to bile duct dilatation

The CBD descends in a groove along the posterior aspect of the pancreatic head and courses toward the duodenum, where it approaches the pancreatic duct near the ampullary region [8]. A commonly used upper limit of normal CBD caliber is approximately 6 mm, although normal diameter may vary with age, prior cholecystectomy, and clinical context [9].

When bile duct dilatation is identified, the first imaging priority is to determine whether true obstruction exists and, if so, its level and cause [10]. Biliary dilatation may be separated into obstructive and non-obstructive causes [11]. Obstructive causes include stones, malignant neoplasms, post-inflammatory or inflammatory strictures, biliary parasites, and extrinsic compression by adjacent structures or lymphadenopathy. Non-obstructive causes include congenital cystic dilatation, like choledochal cysts and Caroli disease, intraductal papillary neoplasm of the bile duct, aging, and post-cholecystectomy state. Primary sclerosing cholangitis and other chronic cholangiopathies may present with both strictures and ductal dilatation [9].

### Preferred imaging strategy

The patient should be evaluated using MRCP combined with contrast-enhanced MRI for ductal mapping, mural characterization, diffusion assessment, and evaluation of periductal soft tissues and vascular relationships [12]. Multiphase CT serves as a complementary modality, particularly for assessing extrinsic compression, vascular anatomy, and staging when malignancy is suspected [13]. The complementary roles of each imaging modality are summarized in Table 1.

**Table 1:** Imaging strategy and technical rationale for biliary stricture evaluation

Imaging Modality	Core diagnostic contribution	Key clinical and technical application
Multiphase CT	Evaluation of extrabiliary disease and whole-body staging	Detection of extrinsic compression (e.g., lymphadenopathy, masses); vascular anatomy and encasement; assessment of regional and distant metastases; preoperative staging and resectability assessment
MRCP	Noninvasive, high-resolution depiction of the biliary tree	Comprehensive ductal mapping; characterization of stricture morphology (length, margins, tapering); detection of intraductal filling defects (e.g., stones, debris); evaluation of upstream dilatation
Contrast-Enhanced MRI (including DWI ± hepatobiliary phase)	Multiparametric tissue characterization and assessment of local tumor extent	Evaluation of mural thickening and enhancement patterns; detection of diffusion restriction; assessment of periductal infiltration and vascular involvement; hepatobiliary phase assessment of functional impairment and subtle lesions

Diffusion-weighted imaging (DWI) with apparent diffusion coefficient (ADC) mapping contributes to mural characterization. However, several important pitfalls must be recognized. Malignant strictures typically demonstrate marked diffusion restriction (low ADC values), while active inflammatory conditions, including IgG4-related cholangitis, PSC dominant strictures, post-procedural changes, and infectious cholangitis may also show restricted diffusion, substantially limiting specificity [14]. Biliary stents introduce susceptibility artifacts that degrade DWI quality in the vicinity of the stricture. When DWI findings are equivocal, correlation with enhancement kinetics, clinical history, and serologic markers is essential [15].

## Morphological subtypes and imaging features of CCA

**Fig. 1:**
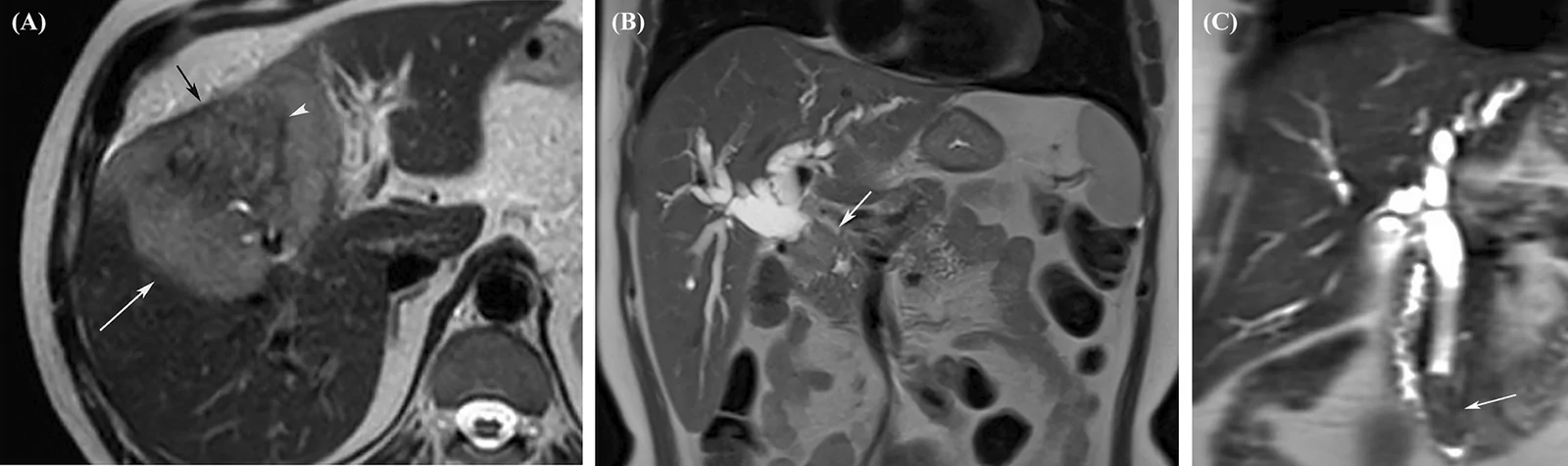
**a**–**c** Morphological classification of cholangiocarcinoma (CCA) on MRI. **A** Mass-forming CCA: heterogeneous mass with a peripheral hypercellular T2-hyperintense rim (white arrow), central fibrous T2-hypointense stroma (arrowhead), and capsular retraction (black arrow). **B** Periductal-infiltrating CCA: periductal soft-tissue thickening with luminal obliteration (arrow) and upstream biliary dilatation.** C** Intraductal-growing CCA: polypoid intraductal lesion (arrow) with marked ductal dilatation and no parenchymal mass

CCA is a primary epithelial malignancy of the biliary tract classified anatomically into intrahepatic (iCCA), perihilar (pCCA), and distal (dCCA) forms, and morphologically, according to the Liver Cancer Study Group of Japan, into mass-forming, periductal-infiltrating, and intraductal-growing growth patterns [4, 6]. These subtypes correlate closely with imaging appearance, tumor spread behavior, and susceptibility to mimicry by benign strictures, making growth pattern recognition an essential framework for interpreting CBD findings. Figure 1 illustrates the morphological classification of cholangiocarcinoma (CCA) on magnetic resonance imaging.

Mass-forming CCA, the predominant subtype of intrahepatic CCA, presents as an exophytic hepatic mass with a characteristic dual-component, “targetoid” architecture expressed consistently across sequences. The viable hypercellular periphery is mildly T2-hyperintense, shows early arterial enhancement and marked diffusion restriction (high signal on high-b images, low ADC); the hypovascular desmoplastic center is mildly T2-hypointense and demonstrates progressive centripetal delayed fill-in. Ancillary features such as capsular retraction, peripheral biliary dilatation, satellite nodules, and occasional vascular invasion further support malignancy [1, 6, 13].

Periductal-infiltrating CCA, the dominant pattern in perihilar and distal CCA, manifests as irregular bile duct wall thickening with longitudinal submucosal spread rather than a discrete mass [1, 4, 6]. In the CBD, it most often produces a long, irregular, asymmetric stricture with shouldered margins, disproportionate upstream biliary dilatation, and peripheral arterial enhancement with progressive centripetal delayed fill-in, reflecting the dense desmoplastic composition [1, 9, 16–18]. Ill-defined periductal soft tissue, vascular encasement of the portal vein or hepatic artery, regional lymphadenopathy, and secondary parenchymal signs of infiltration further support malignancy [4]. MRI with MRCP provides superior delineation of longitudinal tumor extent, which is critical for preoperative staging. No single feature is pathognomonic to differentiate a periductal-infiltrating CCA from a benign biliary stricture. A multiparametric approach is essential, and key distinguishing characteristics are summarized in Table 2.

**Table 2:** Key distinguishing imaging features: cholangiocarcinoma vs. benign biliary strictures

Imaging feature	More common in malignancy (CCA)	More common in benign etiology
Stricture morphology	Long-segment stricture (> 20–30 mm), irregular and asymmetric margins, abrupt luminal narrowing. Note: long-segment stricture may also occur in IgG4-SC.*	Short-segment stricture (< 15 mm), smooth and symmetric margins, with gradual (conical) narrowing
Wall thickening and enhancement	Asymmetric mural thickening with peripheral arterial phase enhancement and progressive delayed centripetal enhancement (fibrotic stroma)	Absent or mild, symmetric mural thickening with minimal or homogeneous enhancement.
Upstream biliary dilatation	Marked and disproportionate upstream ductal dilatation with abrupt transition	Mild to moderate dilatation with gradual transition, proportional to degree of narrowing
Vascular involvement	Encasement, irregular narrowing, or invasion of adjacent vessels (portal vein/hepatic artery); loss of intervening fat planes. Note: vascular encasement may also be seen in IgG4-SC (typically smooth, without true occlusion).*	Preserved vessel caliber and contour; intact perivascular fat planes
Periductal soft tissue	Ill-defined periductal infiltration or mass-like soft tissue extending beyond duct wall. Note: periductal soft tissue also present in IgG4-SC (symmetric, smooth) — pattern and symmetry are key differentiating features.*	Absent or minimal
DWI / ADC	Marked diffusion restriction (low ADC) in a focal or segmental pattern; asymmetric restriction	No restriction, or symmetric mild restriction; note: inflammatory strictures (PSC, IgG4-SC, cholangitis) may also show restriction — specificity is limited
Identifiable cause	Usually absent	Often present (e.g., choledocholithiasis, post-surgical stricture, pancreatitis, IgG4-related disease, parasitic infection)
Temporal evolution	Persistence or interval progression on serial imaging	Resolution or improvement after biliary drainage or anti-inflammatory therapy

*No imaging feature is pathognomonic. IgG4-related sclerosing cholangitis may exhibit long-segment stricture, symmetric mural thickening, vascular encasement, and periductal soft tissue, closely mimicking CCA. Ancillary clinical, serologic, and histologic findings are essential for differentiation

Intraductal-growing CCA is the least common subtype and carries the most favorable prognosis. It appears as an intraluminal polypoid or papillary mass causing focal ductal dilatation with preserved ductal contour, ranging from a localized filling defect to diffuse marked ductectasia without an obvious mass, a spectrum that closely mimics intraductal papillary mucinous neoplasm of the bile duct or primary sclerosing cholangitis [1, 6].

### Hepatobiliary phase imaging

Regardless of subtype, CCA appears markedly hypointense on hepatobiliary phase imaging, reflecting absent hepatocyte uptake of hepatocyte-specific contrast agents [19, 20]. This persistent hypointensity improves lesion conspicuity, facilitates detection of satellite nodules that may be isoattenuating on earlier phases, and delineates the craniocaudal extent of periductal infiltration beyond the primary stricture, a function particularly valuable in the periductal-infiltrating subtype, where arterial enhancement may be minimal and T2WI signal changes subtle [21].

Of the two available hepatobiliary agents, gadoxetate disodium (Primovist/Eovist; hepatic extraction fraction ~ 50%) yields a more pronounced hepatobiliary phase achievable earlier (15–20 min) with superior liver-to-lesion contrast compared with gadobenate dimeglumine (MultiHance; ~3–5%; requiring 40–120 min), making it the preferred agent when hepatobiliary phase assessment is the primary clinical objective [20]. Its principal limitation is transient severe motion artifact during the arterial phase, which may compromise dynamic characterization; gadobenate dimeglumine is preferred in this setting or when high-fidelity arterial phase assessment is the primary goal. For example, when distinguishing CCA from HCC or hypervascular metastases [20]. Agent selection should ultimately be guided by the primary clinical question and institutional protocol.

## Pictorial differential diagnosis: key mimickers of CBD cholangiocarcinoma

To facilitate pattern recognition, each mimicker in this review is also classified according to the CCA morphological subtype it most closely resembles: periductal-infiltrating, intraductal-growing, or extrinsic compression. The main etiological categories of CBD cholangiocarcinoma mimickers are summarized in Table 3. Identifying the dominant CCA growth pattern on imaging is the first step toward generating a focused and clinically relevant differential diagnosis.

**Table 3:** Etiological classification of common bile duct (CBD) cholangiocarcinoma mimickers

Etiological category	Representative pathologies and diagnostic entities	CCA subtype most closely mimicked
Intraductal Obstructive Causes	Choledocholithiasis; intraductal or periampullary neoplasms (e.g., villous adenoma); parasitic infestation (e.g., liver flukes, Fasciola hepatica); Lemmel syndrome (periampullary duodenal diverticulum)	*Intraductal-growing*
Inflammatory and Immune-Mediated Strictures	IgG4-related sclerosing cholangitis (IgG4-SC); primary sclerosing cholangitis (PSC); benign inflammatory strictures with smooth, gradual tapering	*Periductal-infiltrating*
Infectious and Systemic Granulomatous Diseases	Paracoccidioidomycosis; sarcoidosis; other granulomatous infections	*Periductal-infiltrating*
Neoplastic Mimickers and Extrinsic Compression	Lymphoma (primary or secondary hilar involvement); metastatic lymphadenopathy causing extrinsic biliary compression; biliary embryonal rhabdomyosarcoma	*Intraductal-growing (rhabdomyosarcoma); Periductal-infiltrating / Extrinsic compression (lymphoma*,* metastatic nodes)*
Post-Surgical and Adjacent Inflammatory Conditions	Iatrogenic biliary strictures (e.g., post-cholecystectomy, biliary-enteric anastomosis); xanthogranulomatous cholecystitis (XGC)	*Periductal-infiltrating*
Congenital and Non-Obstructive Ductal Abnormalities	Choledochal cysts (Todani classification); Caroli disease	*Intraductal-growing*
Vascular-Related Entities (Portal Biliopathy and Arterial Compression)	Portal biliopathy / portal cavernoma cholangiopathy; extrinsic compression by adjacent vessels (e.g., right hepatic artery)	*Extrinsic compression*

### Obstructive intraductal causes

Obstructive intraductal mimickers represent the most frequent mimickers of CCA, as they may produce ductal narrowing and upstream dilatation without true mural infiltration. They are characterized by luminal filling defects or extrinsic compression, typically with preservation of ductal wall architecture and absence of infiltrative enhancement [5, 6]. In contrast, CCA is primarily a disease of the ductal wall, presenting with asymmetric thickening, progressive enhancement, and frequent periductal extension [4, 22]. Integration of MRCP morphology with contrast-enhanced MRI and CT findings allows confident differentiation in most cases [14, 18].

#### Choledocholithiasis

**Fig. 2:**
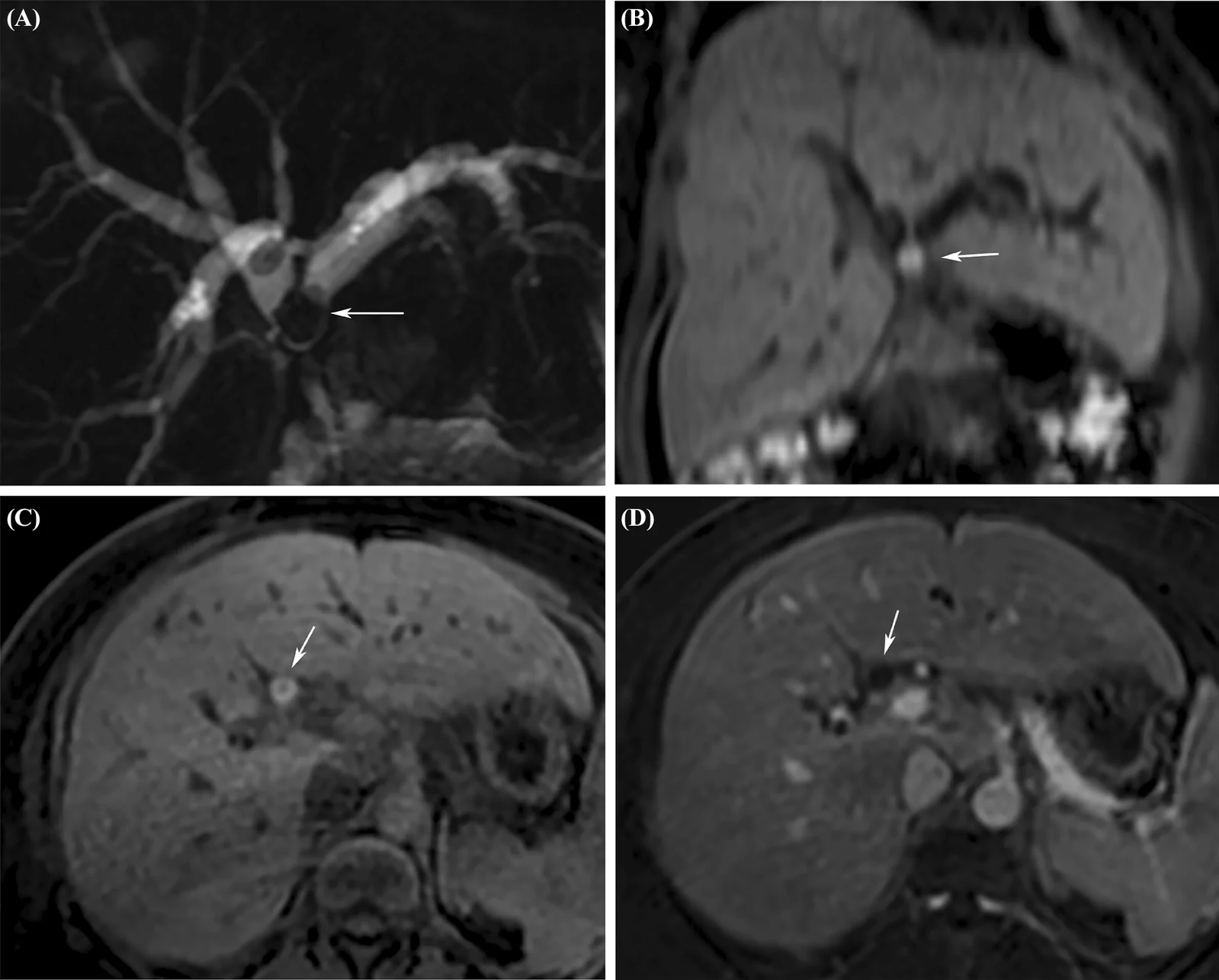
Choledocholithiasis. **A** MR cholangiography demonstrates intrahepatic biliary ductal dilatation and a filling defect on the confluence of the right and left hepatic ducts (arrow). **B**,** C** Pre-contrast reconstructed coronal and axial T1-weighted image show a spontaneously hyperintense stone in the proximal CBD (arrows). **D** Post-contrast subtraction image demonstrates no enhancement (arrow), supporting the diagnosis of a pigmented stone. *Key features*: a meniscus of high-signal bile surrounding a smoothly marginated hypointense defect, without mural thickening or a soft-tissue mass, favors a calculus over a neoplasm

Choledocholithiasis is the most common benign cause of CBD obstruction and occurs in approximately 16% of patients with cholelithiasis. On MRCP, stones appear as well-defined intraluminal filling defects with upstream ductal dilatation (Fig. 2) [9]. On MRI, they are typically hypointense on T2-weighted images and do not enhance [23]. CT may demonstrate a hyperattenuating focus depending on the stone composition. Impacted stones may induce reactive mural thickening, occasionally simulating an infiltrative stricture [9]. However, smooth, short-segment narrowing, absence of asymmetric wall thickening or delayed enhancement, and visualization of bile surrounding the lesion favor a benign diagnosis, whereas CCA shows irregular strictures, asymmetric mural thickening, and periductal soft-tissue infiltration [5].

#### Mirizzi syndrome

**Fig. 3:**
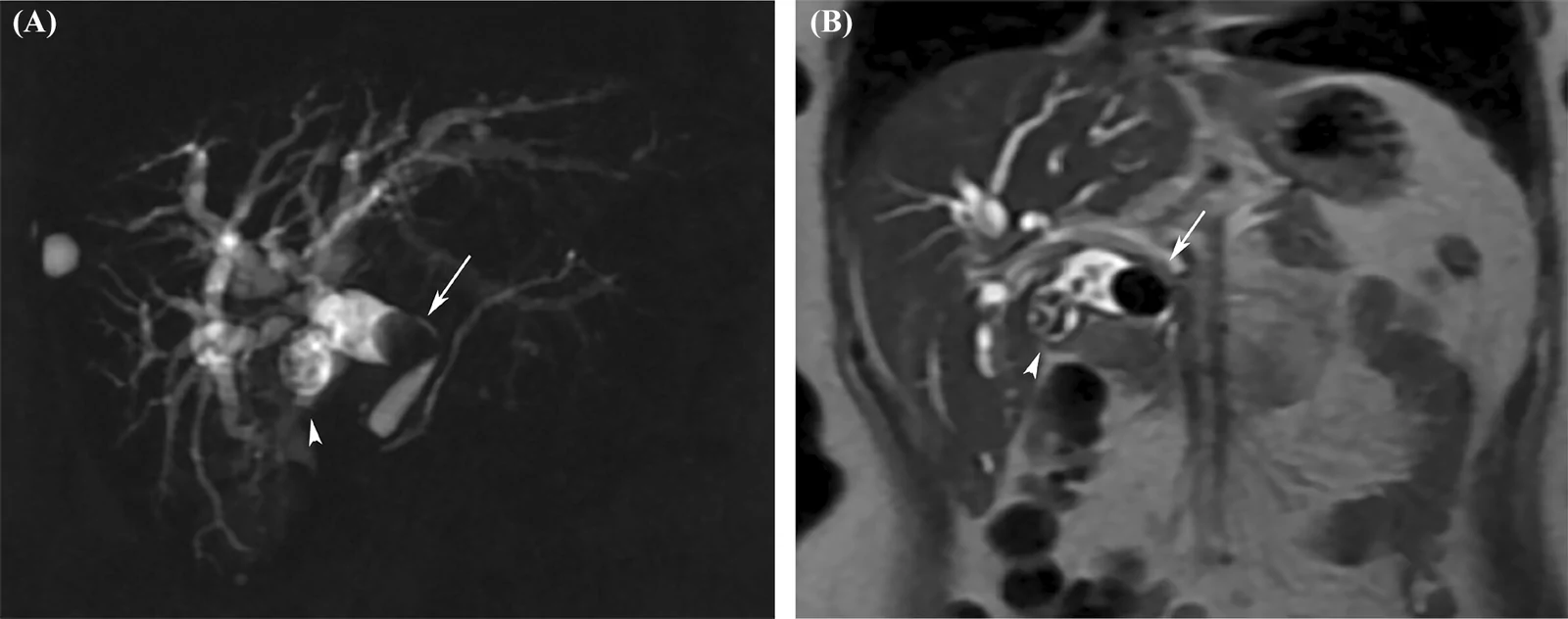
Mirizzi syndrome. **A**,** B** Coronal heavily T2-weighted MRCP and coronal T2-weighted image show an impacted T2-hypointense calculus in the cystic duct/gallbladder neck (arrow) compressing the adjacent common hepatic duct, with upstream intrahepatic biliary dilatation, a normal-caliber distal CBD and a contracted, thick-walled gallbladder (arrowhead) with pericholecystic inflammation. *Key features*: the triad of an impacted cystic duct/gallbladder neck stone, smooth extrinsic CHD narrowing at the same level, and proximal-only dilatation distinguishes Mirizzi from choledocholithiasis, in which the stone lies within the CBD

Mirizzi syndrome is a rare complication of gallstone disease, that occurs in approximately 0.7–1.4% of patients with cholelithiasis. It is caused by extrinsic compression of the common hepatic duct or CBD by an impacted stone in the cystic duct or gallbladder neck. Patients usually present with abdominal pain, jaundice, and inflammatory symptoms. MRCP is the most sensitive imaging modality, demonstrating the impacted stone, level of obstruction, and upstream dilatation. CT and MRI may show gallbladder wall thickening and pericholecystic inflammatory changes (Fig. 3) [24]. The key feature is extrinsic compression by a stone rather than an intraductal or infiltrative lesion. Absence of an enhancing mass and lack of vascular involvement support a benign diagnosis [5].

#### Fasciola hepatica

**Fig. 4:**
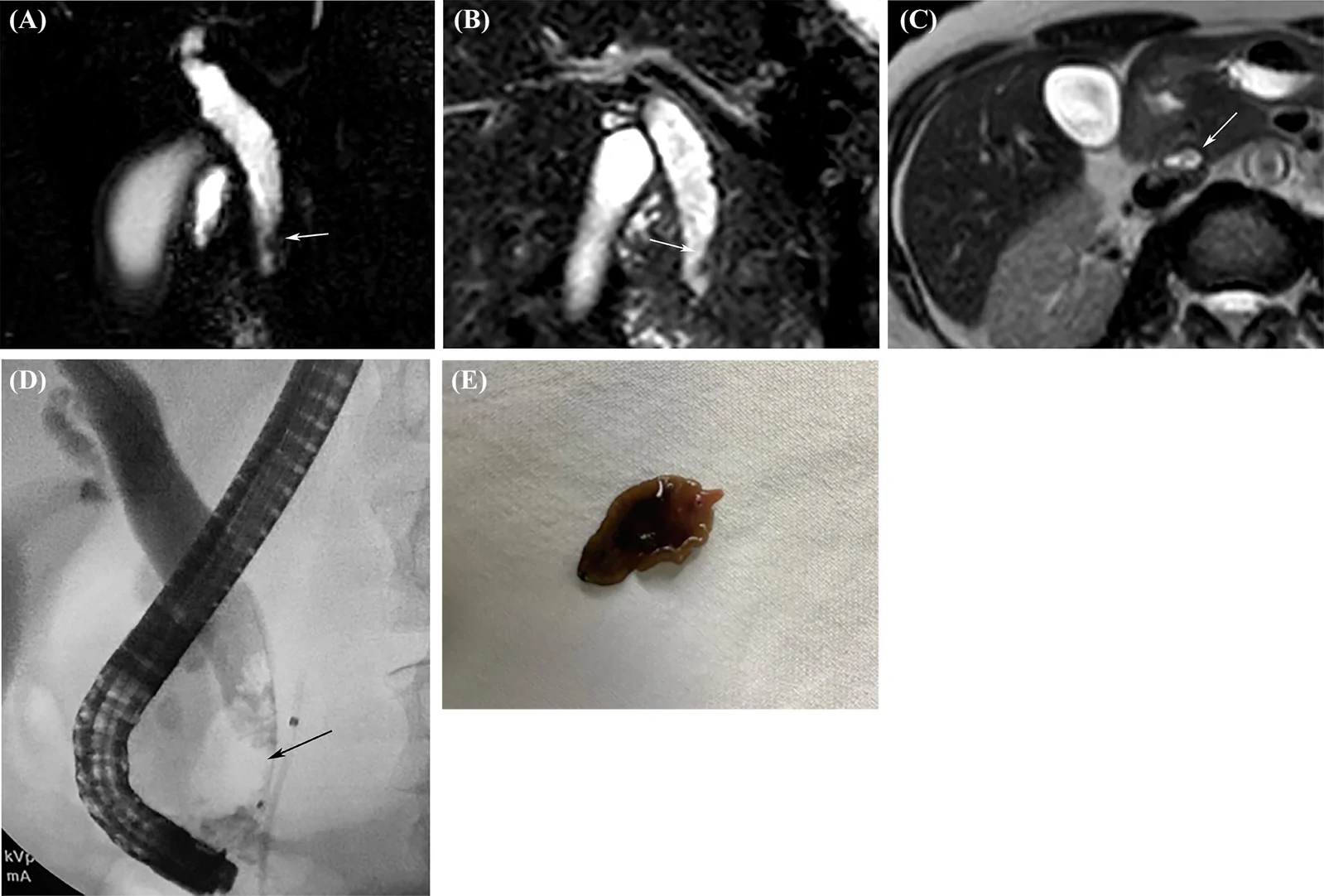
Fasciola hepatica **A**,** B** MR cholangiography and coronal T2-weighted image show a linear / leaf-shaped and mildly irregular filling defect in the distal common bile duct (arrow). **C** Axial T2-weighted image demonstrates a linear hypointense structure within the distal common bile duct (arrow). **D** Cholangiographic image demonstrates the distal common bile duct filling defect (arrow). **E** Extracted Fasciola hepatica specimen following the procedure. *Key features*: a mobile, linear or leaf-shaped (often crescentic) intraductal filling defect, distinct from the rounded, dependent appearance of a calculus, without mural thickening is highly suggestive of biliary fascioliasis, particularly with supporting clinical and epidemiologic context (eosinophilia, watercress exposure)

Fasciola hepatica infection is an uncommon cause of biliary obstruction outside endemic regions but should be considered in the appropriate clinical context. Patients may present with abdominal pain, cholestasis, or cholangitis. On MRCP, characteristic linear or leaf-shaped intraluminal filling defects are seen, sometimes with subtle positional change (Fig. 4). MRI may demonstrate associated ductal dilatation and variable mural thickening, which can mimic malignant strictures [25]. Elongated intraductal structures, absence of a discrete enhancing mass, and lack of progressive delayed enhancement favor a parasitic etiology. CCA typically manifests as fixed strictures with infiltrative enhancement [5].

#### Periampullary villous adenomas

**Fig. 5:**
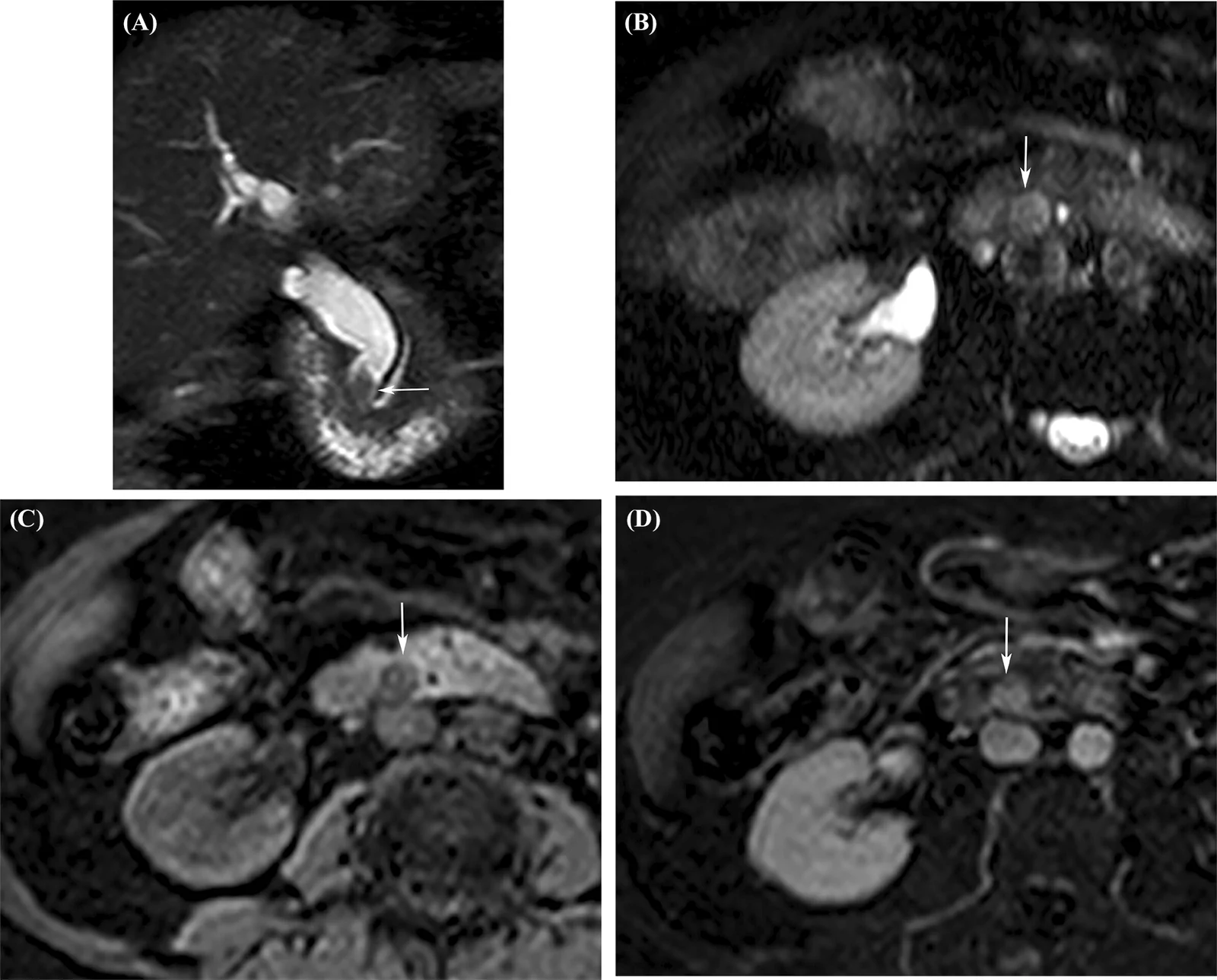
Periampullary villous adenoma. **A**,** B** Coronal and axial fat-suppressed T2-weighted image demonstrate a nodular filling defect in the distal common bile duct (arrows), associated with upstream biliary dilatation. **C** Pre-contrast T1-weighted image demonstrates low signal intensity of the lesion (arrow). **D** Portal venous phase subtraction image demonstrates contrast enhancement of the lesion (arrow). *Key features*: a well-circumscribed polypoid periampullary lesion with smooth margins, intact duct walls, and gradual upstream ductal dilatation favors a benign or low-grade neoplasm over periampullary adenocarcinoma; given the substantial risk of harboring or progressing to invasive carcinoma, endoscopic biopsy and histopathologic correlation are required

Periampullary villous adenomas are rare benign epithelial tumors that may obstruct the distal bile duct and pancreatic duct, producing upstream biliary and/or pancreatic duct dilatation [9, 26]. Patients typically present with jaundice or pancreatitis. On CT and MRI, these lesions may appear as well-defined polypoid or lobulated masses at the ampulla, although small lesions can be occult on cross-sectional imaging [8, 26]. MRCP shows upstream ductal dilatation with smooth distal narrowing (Fig. 5). Regular ductal margins without infiltrative wall thickening or periductal extension favor a benign etiology. Lack of delayed enhancement further supports benignity, whereas irregular margins and progressive enhancement raise suspicion for malignancy [5].

#### Lemmel syndrome

**Fig. 6:**
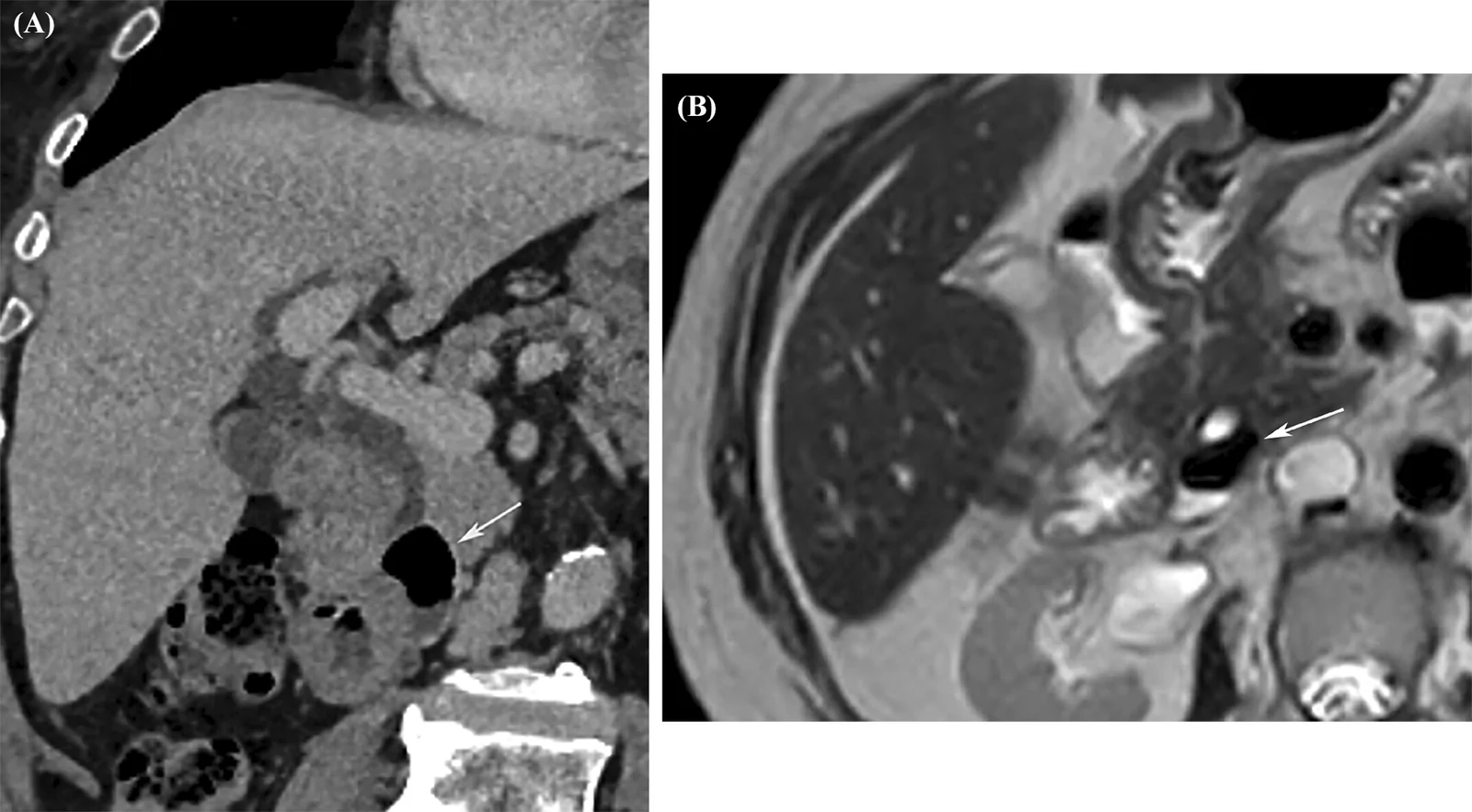
Lemmel syndrome **A** Coronal portal venous phase CT reconstruction demonstrates a gas-filled diverticulum (arrow) arising from the second portion of the duodenum that compresses the distal common bile duct and causes mild upstream biliary dilatation. **B** Axial T2-weighted image shows the periampullary diverticulum (arrow) compressing the distal common bile duct, displaced slightly anteriorly. *Key features*: a juxtapapillary diverticulum containing air, fluid, oral contrast, or debris in direct contact with the distal CBD, together with upstream dilatation and exclusion of other obstructive causes, establishes the diagnosis. Recognition matters because the diverticulum is commonly mistaken for a pancreatic head cystic lesion or periampullary abscess

Lemmel syndrome, caused by a periampullary duodenal diverticulum, is an uncommon source of distal biliary obstruction. Patients may present with jaundice, cholestasis, and abdominal discomfort. On CT, a periampullary sac-like structure containing air or fluid may be identified adjacent to the duodenum, compressing the distal CBD (Fig. 6). MRI and MRCP demonstrate smooth distal narrowing with upstream dilatation, without intraductal mass or irregular mural thickening [27]. Recognition of the diverticulum and preserved ductal wall architecture favor a benign process. CCA typically presents with irregular strictures and progressive enhancement [5].

### Inflammatory and immune-mediated strictures

Inflammatory and immune-mediated biliary strictures primarily involve the ductal wall and may closely mimic CCA [5, 28]. They typically present with smooth, symmetric thickening and gradual tapering, whereas CCA is characterized by irregular narrowing, asymmetric wall thickening, progressive enhancement, and periductal extension [5, 18]. Combined assessment with MRCP and contrast-enhanced MRI/CT is essential for differentiation [18].

#### Primary sclerosing cholangitis (PSC)

**Fig. 7:**
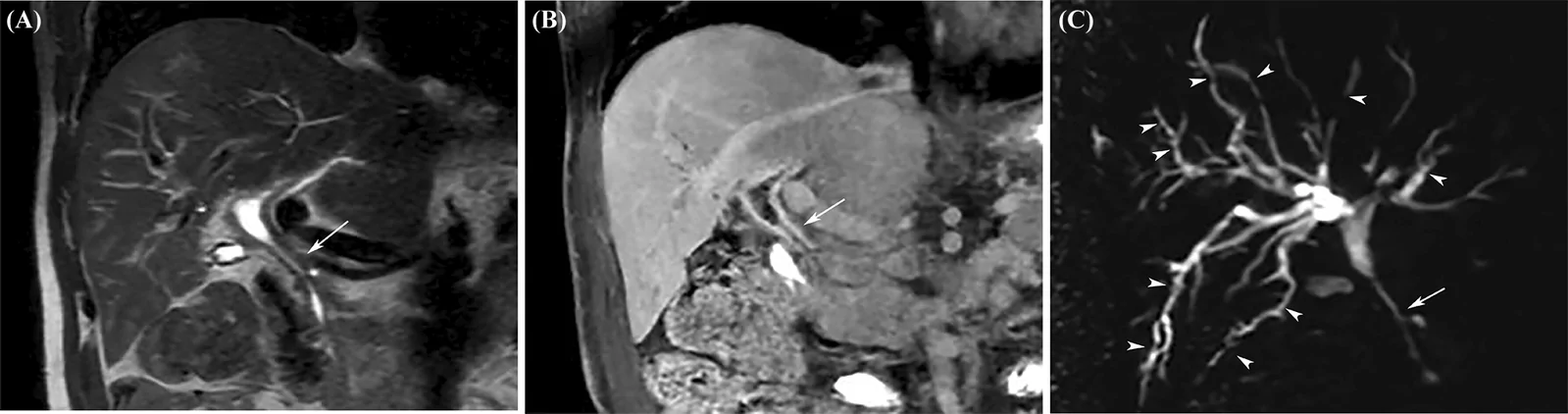
Primary sclerosing cholangitis (PSC). **A** Coronal T2-weighted image demonstrates diffuse wall thickening of the common bile duct (arrow), with gradual narrowing involving its mid-to-distal portion. **B** Post-contrast coronal T1-weighted image shows enhancement of the thickened common bile duct wall (arrow). **C** MR cholangiography demonstrates gradual narrowing of the mid-to-distal common bile duct (arrow), as well as multifocal alternating mild strictures and dilatations involving the intrahepatic bile ducts (arrowheads) consistent with primary sclerosing cholangitis. *Key features*: multifocal short-segment strictures with intervening normal or mildly dilated segments, peripheral pruning, and absence of a dominant mass favor PSC over secondary sclerosing cholangitis or cholangiocarcinoma; a new high-grade dominant stricture, especially with mural thickening or progressive enhancement, should raise concern for superimposed cholangiocarcinoma

PSC is a chronic cholestatic disease affecting intra- and extrahepatic bile ducts, strongly associated with inflammatory bowel disease [29, 30]. MRCP demonstrates multifocal short-segment strictures alternating with normal or mildly dilated segments (Fig. 7). The AASLD recommends using specific terms such as “stenosis,” “stricture,” and “dilatation” rather than imprecise descriptions such as “beaded”. Contrast-enhanced MRI may show mild biliary wall thickening and mural enhancement, with progressive ductal obliteration as fibrosis advances [30].

Dominant strictures develop in up to 45% of patients and may mimic periductal-infiltrating CCA, particularly when asymmetric [30]. However, differentiating benign from malignant strictures in PSC remains challenging, MRI/MRCP shows suboptimal sensitivity (43–50%) and specificity (70–76%) for ductal CCA detection [31]. Features that favor PSC include multifocal distribution and absence of a discrete mass. Focal, irregular strictures with asymmetric thickening and progressive enhancement are more characteristic of CCA [12]. PSC carries a lifetime CCA risk of up to 20%, with an annual incidence of 0.6–1.5% [30, 32]. Surveillance with MRI/MRCP combined with CA 19 − 9 every 6–12 months is recommended [30, 33].

#### IgG4-related sclerosing cholangitis (IgG4-SC)

**Fig. 8:**
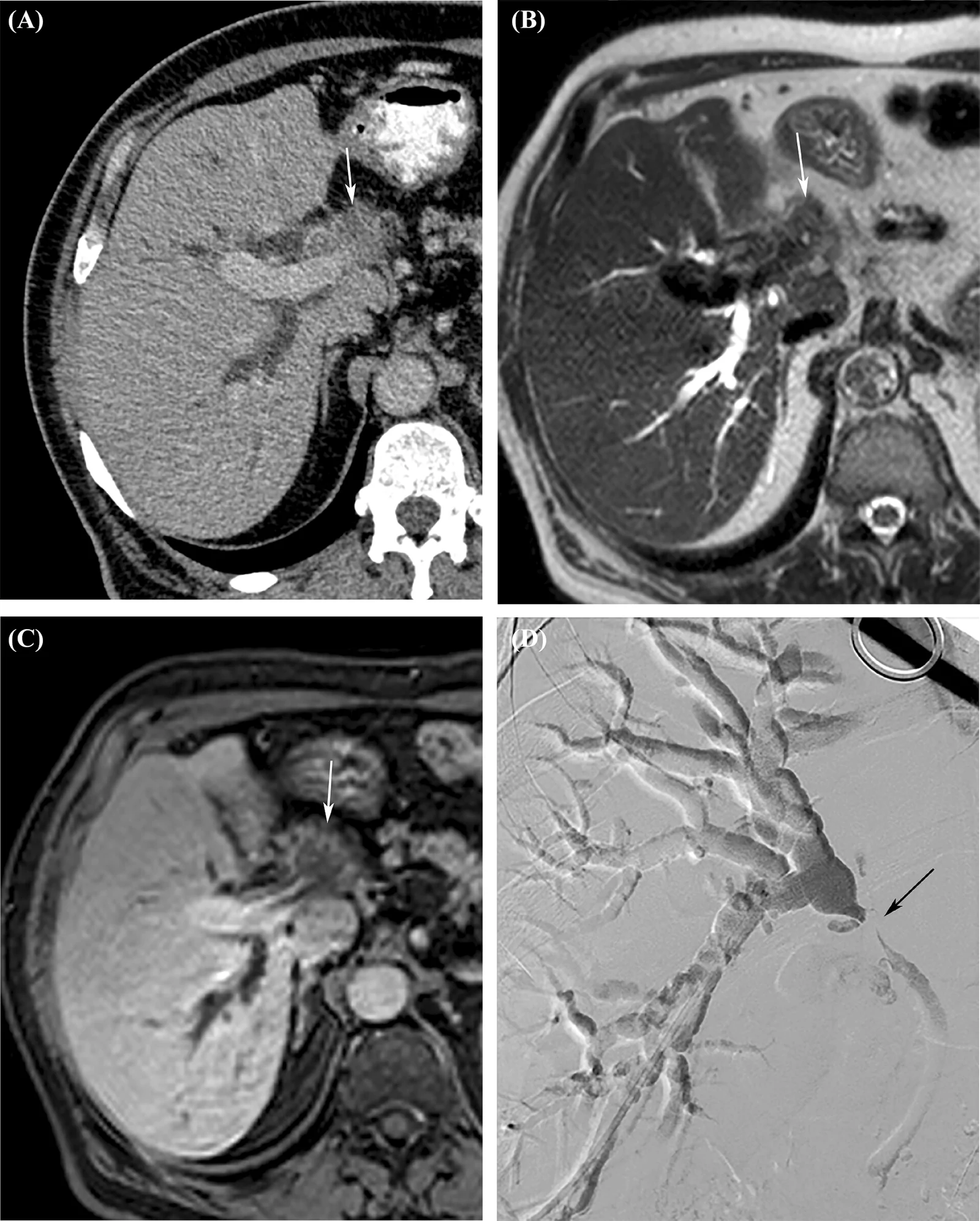
IgG4-related sclerosing cholangitis (IgG4-SC). **A** Portal venous phase CT image demonstrates irregular and ill-defined soft tissue thickening at the hepatic hilum (arrow), involving the mid-to-upper portion of the common bile duct associated with upstream intrahepatic biliary dilatation. **B** Axial T2-weighted image shows infiltrative-appearing soft tissue thickening surrounding the common bile duct (arrow). **C** Post-contrast portal venous phase MR image demonstrates enhancement of the infiltrative hilar soft tissue thickening (arrow). **D** MR cholangiography shows gradual narrowing of the upper common bile duct (arrow) with upstream intrahepatic biliary dilatation. *Key features*: long-segment, symmetric, concentric thickening with smooth margins and homogeneous enhancement disproportionate to luminal narrowing favors IgG4-SC over cholangiocarcinoma, in which thickening is typically eccentric, irregular, shouldered, and progressively enhancing. Synchronous autoimmune pancreatitis or other IgG4-related manifestations, elevated serum IgG4, and steroid responsiveness support the diagnosis, which is formally established by HISORt or ICDC criteria rather than imaging alone

IgG4-SC is a fibroinflammatory biliary disease that frequently involves the hilar and extrahepatic ducts and may simulate CCA. CT and MRI show long-segment, symmetric, circumferential wall thickening with smooth margins and preserved lumen. Enhancement is typically homogeneous, without the progressive delayed pattern characteristic of CCA (Fig. 8). MRCP demonstrates long, continuous strictures without beading. Symmetric thickening, long-segment involvement, and lumen preservation favor IgG4-SC. CCA typically presents with short, irregular strictures, abrupt narrowing, and asymmetric thickening. Associated extrabiliary findings, particularly autoimmune pancreatitis, strongly support the diagnosis [28, 34]. The diagnosis of IgG4-SC is supported by the HISORt criteria (Histology, Imaging, Serology, Other organ involvement, and Response to steroid therapy), which integrate imaging with serologic and histopathologic parameters [35]. Elevated serum IgG4 levels and favorable response to corticosteroid therapy further support the diagnosis [36]. Nevertheless, serum IgG4 lacks sufficient specificity as an isolated biomarker, as elevated levels may also be observed in approximately 10% of patients with CCA [37]. Therefore, a monitored steroid trial with interval imaging represents an accepted diagnostic approach in carefully selected cases with low suspicion for malignancy [35].

#### Benign inflammatory strictures (gradual tapering pattern)

**Fig. 9:**
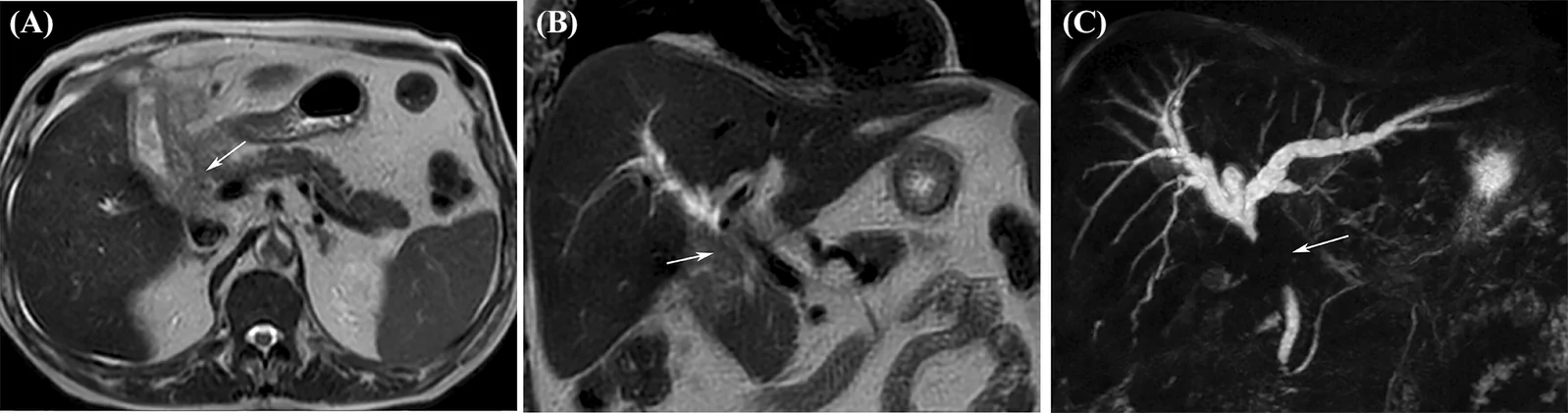
Benign inflammatory biliary stricture (gradual tapering pattern). **A** Axial T2-weighted image demonstrates gallbladder wall thickening and adjacent inflammatory soft tissue (arrow), suggestive of cholecystitis. The inflammatory changes extend to the hepatic hilum, involving the upper portion of the common bile duct. **B** Coronal T2-weighted image shows hilar soft tissue thickening surrounding the common bile duct (arrow). **C** MR cholangiography demonstrates gradual tapering of the mid-to-upper common bile duct (arrow), associated with upstream biliary dilatation. *Key features*: smooth, concentric tapering with symmetric margins and dilatation proportionate to the degree of narrowing favors a benign etiology, most commonly chronic pancreatitis, prior choledocholithiasis, post-operative injury, or IgG4-SC, over a malignant stricture, which is typically abrupt, asymmetric, and shouldered with disproportionate upstream dilatation. Because imaging overlap with malignancy exists, indeterminate strictures warrant EUS, ERCP with brush cytology or intraductal biopsy, and serologic follow-up (CA 19-9, IgG4)

Benign inflammatory strictures result from iatrogenic, inflammatory, or ischemic injury [34, 38]. MRCP shows smooth, short-segment narrowing with gradual tapering and mild upstream dilatation. MRI and CT typically demonstrate preserved ductal architecture without mass or significant periductal soft tissue (Fig. 9) [12, 38]. Smooth tapering with symmetric narrowing favors benignity, while abrupt, irregular narrowing with asymmetric thickening and progressive enhancement suggests CCA [12, 18]. A long, smooth, tapering stricture, may be observed in benign inflammatory conditions but does not reliably exclude malignancy; therefore, tissue sampling should be considered when diagnostic uncertainty persists [12, 39, 40].

### Infectious and systemic granulomatous disease

Granulomatous diseases may involve the biliary tract through ductal infiltration or extrinsic compression, producing strictures or mass-like lesions that mimic CCA [5, 12]. Compared with CCA, benign granulomatous processes more often show smooth margins, symmetric thickening, and lack of vascular invasion [12, 39].

#### Biliary sarcoidosis

**Fig. 10:**
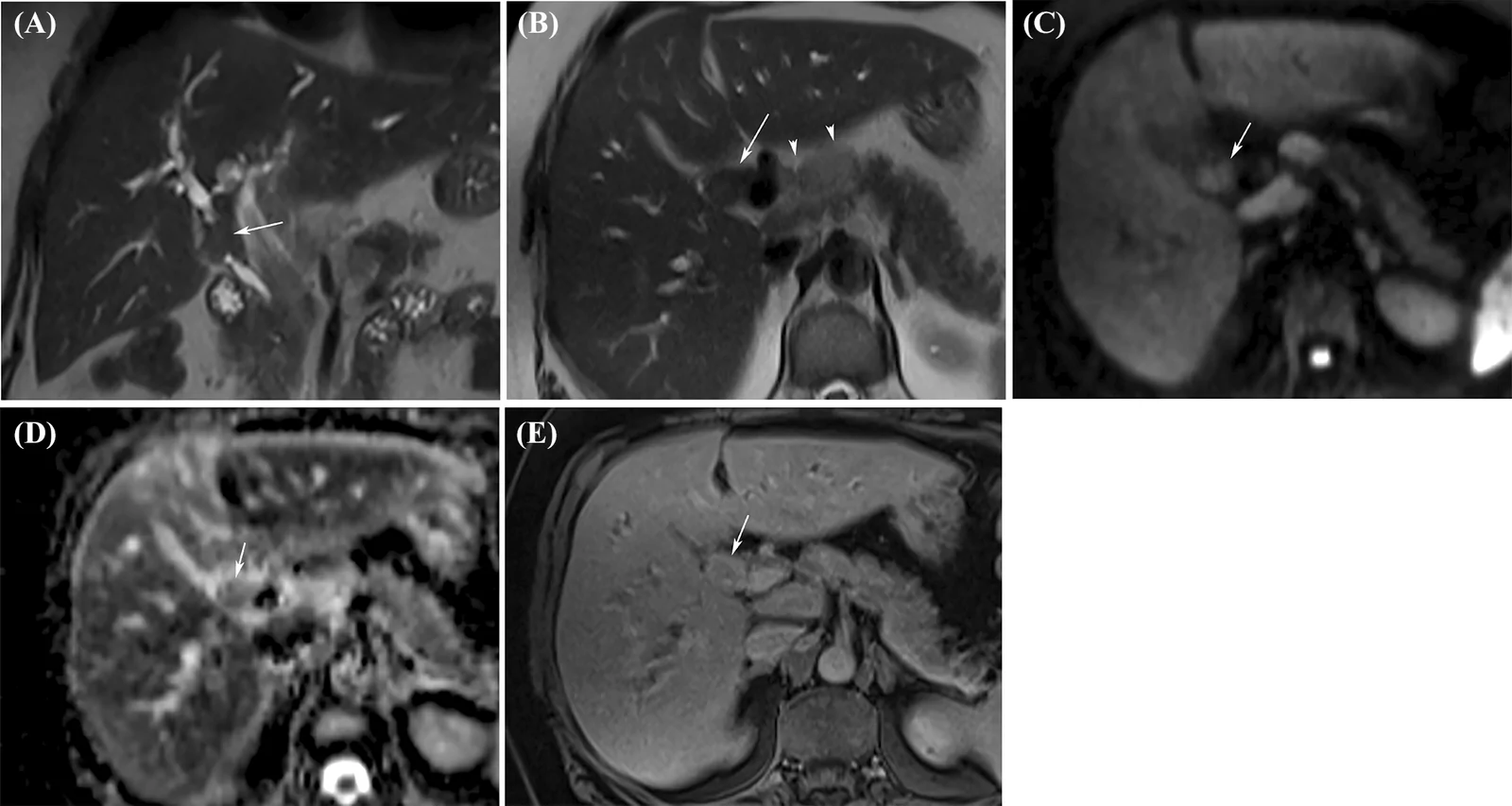
Biliary sarcoidosis. **A** Coronal T2-weighted image demonstrates irregular soft tissue thickening involving the upper portion of the common bile duct (arrow). **B** Axial T2-weighted image shows infiltrative soft tissue surrounding the common bile duct (arrow), associated with periportal lymphadenopathy (arrowheads). **C**, **D** Diffusion-weighted imaging and ADC map demonstrates diffusion restriction within the infiltrative soft tissue (arrow). **E** Post-contrast image demonstrates enhancement of the infiltrative soft tissue (arrow). *Key features*: hepatobiliary sarcoidosis mimics both cholangiocarcinoma and primary scerosing cholangitis and lacks any pathognomonic imaging feature; consider it when these patterns occur with bilateral hilar/mediastinal lymphadenopathy, perilymphatic pulmonary micronodularity, splenic/hepatic microgranulomas, or known multisystem sarcoidosis. Histopathologic confirmation (non-caseating granulomas with exclusion of infection and malignancy) is required

Sarcoidosis is a multisystem granulomatous disease that may involve the liver and bile ducts, with hepatic granulomas found in 20–30% of systemic cases [41, 42]. MRCP and MRI may demonstrate focal hilar strictures, ductal irregularity, or mass-like periductal encasement, closely simulating perihilar CCA (Fig. 10) [6, 10]. Obstructive jaundice may result from granulomatous infiltration of the ductal wall or extrinsic compression by hilar lymphadenopathy [5].

Systemic findings, such as bilateral hilar lymphadenopathy, pulmonary micronodules, cutaneous or ocular involvement and absence of metastatic disease favor sarcoidosis [43]. CCA typically presents with irregular strictures, asymmetric wall thickening, and progressive enhancement. Imaging findings are nonspecific, and histologic confirmation is often required [5].

#### Paracoccidioidomycosis (PCM)

Paracoccidioidomycosis is a systemic granulomatous fungal infection endemic to Latin America that may disseminate to multiple organs, including the liver and biliary tract [44, 45]. Both clinical forms can mimic several infectious and non-infectious disorders, including cancer, sarcoidosis, tuberculosis, and histoplasmosis [44].

**Fig. 11:**
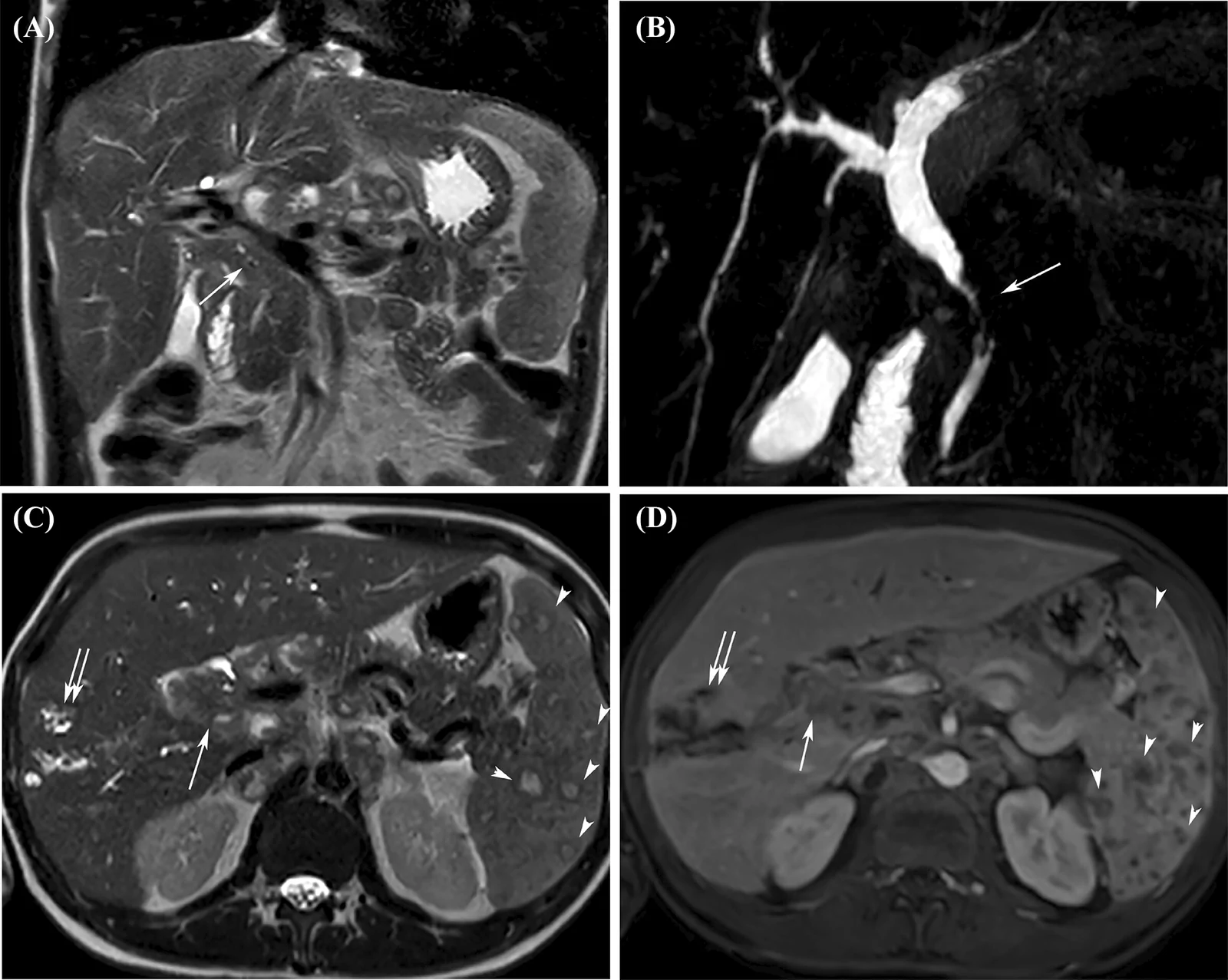
Hepatobiliary paracoccidioidomycosis. **A** Coronal T2-weighted image demonstrates hilar soft tissue thickening involving the mid-to-distal portion of the common bile duct (arrow). **B** MR cholangiography shows gradual tapering of the mid common bile duct (arrow) with upstream biliary dilatation. **C** Axial T2-weighted image demonstrates hilar soft tissue thickening (arrow), associated with hepatic abscesses (double arrow) in the right hepatic lobe and small splenic abscesses (arrowheads). **D** Post-contrast portal venous phase MR image shows enhancement of the hilar soft tissue thickening (arrow), as well as hepatic abscesses (double arrow) and splenic abscesses (arrowheads). Key features: infiltrative hilar disease mimicking cholangiocarcinoma should prompt consideration of paracoccidioidomycosis in patients from endemic regions of Latin America (particularly rural Brazil) with suggestive systemic findings, such as disseminated lymphadenopathy, hepatosplenic microabscesses, adrenal enlargement, pulmonary “reversed halo” or interstitial disease, or mucocutaneous ulcerative lesions. Definitive diagnosis requires microbiologic or histopathologic confirmation (characteristic “mariner’s wheel” Paracoccidioides yeast) with serology and antifungal response

Biliary obstruction may result from ductal infiltration or extrinsic compression by lymphadenopathy, and CT/MRI may show strictures, wall thickening, or mass-like lesions mimicking CCA (Fig. 11) [5, 6]. Diffuse lymphadenopathy and associated systemic findings, such as pulmonary nodules, mucocutaneous lesions, adrenal involvement, favor PCM [44]. CCA is characterized by irregular, asymmetric thickening, progressive enhancement, and vascular invasion [12].

### Neoplastic mimickers and extrinsic compression

#### Biliary embryonal rhabdomyosarcoma

**Fig. 12:**
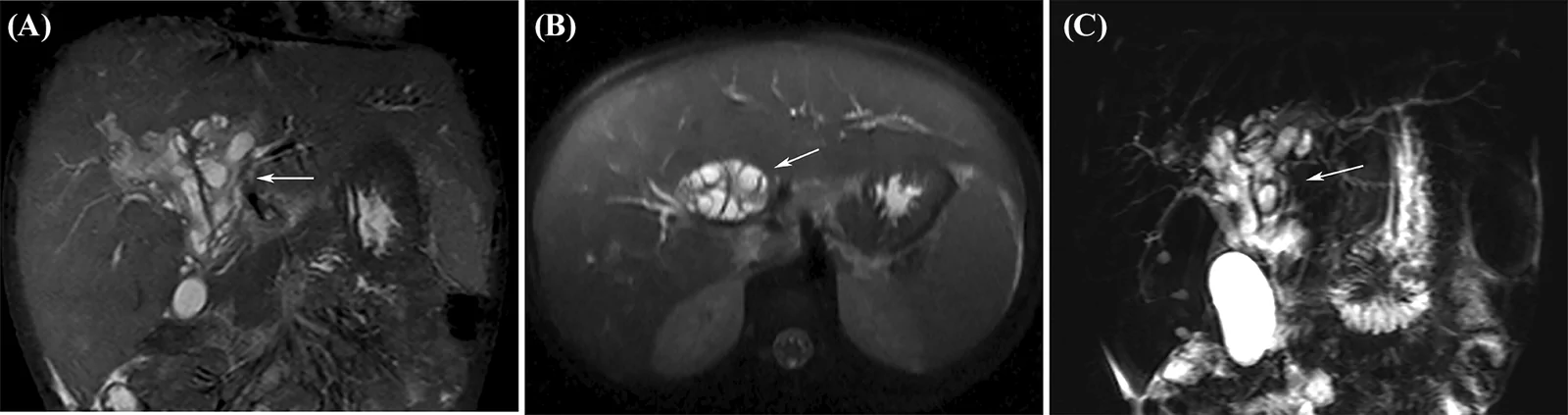
Biliary embryonal rhabdomyosarcoma. **A**, **B** Coronal and axial fat-suppressed T2-weighted image demonstrate marked irregular heterogeneous expansive soft tissue thickening involving the common bile duct and the right and left hepatic ducts, with interspersed cystic-appearing areas (arrow). **C** MR cholangiography demonstrates extensive irregular involvement of the biliary ducts by the expansive lesion (arrow). *Key features*: a polypoid, grape-like intraductal mass producing expansile, fusiform biliary dilatation in a child or adolescent (typically 2–6 years) strongly favors biliary embryonal rhabdomyosarcoma, the most common pediatric biliary malignancy, and the patient’s age effectively excludes intraductal-growing cholangiocarcinoma, an entity of older adults. Diagnosis requires histopathology

Biliary embryonal rhabdomyosarcoma is a rare pediatric tumor of the extrahepatic bile ducts and the most common biliary malignancy in children [46]. Imaging shows an intraductal mass with fusiform ductal dilation, abrupt cutoff, and upstream ectasia. On MRI, the tumor is T1 hypointense, T2 hyperintense, with heterogeneous enhancement, and MRCP reveals an intraluminal filling defect (Fig. 12) [5, 46]. The intraductal polypoid pattern may mimic intraductal-growing CCA. However, pediatric age and expansile intraluminal growth favor rhabdomyosarcoma [5, 6].

#### Lymphoma

**Fig. 13:**
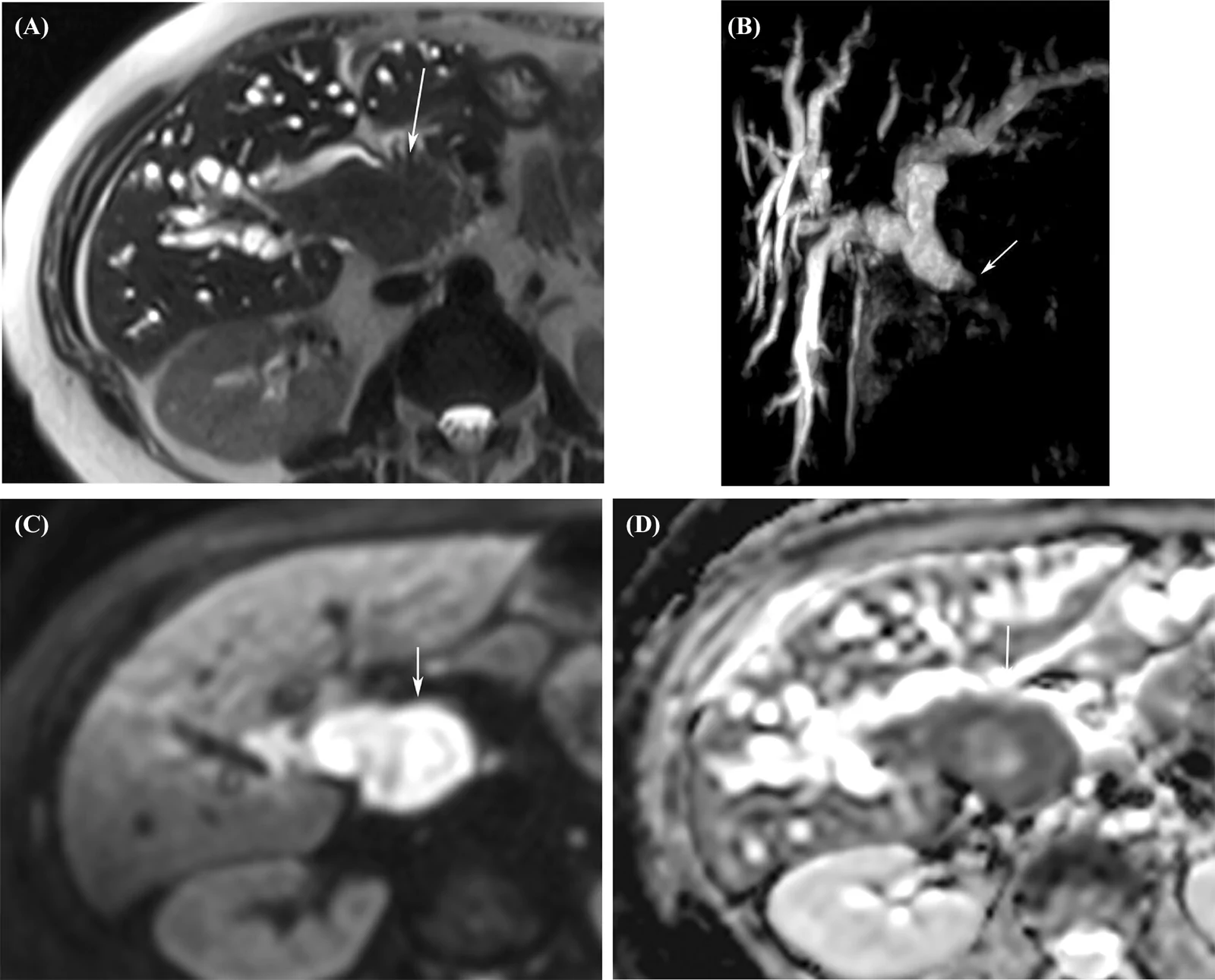
Lymphoma **A** Axial T2-weighted image demonstrates an expansive hilar lesion involving the distal portion of the common bile duct (arrow) with upstream biliary dilatation. **B** MR cholangiography shows gradual narrowing of the distal common bile duct (arrow) with upstream biliary dilatation. **C**, **D** Diffusion-weighted imaging and ADC map demonstrate diffusion restriction within the hilar lesion (arrow). *Key features*: bulky, homogeneous, T2-isointense periductal soft tissue with marked uniform diffusion restriction, homogeneous mild-to-moderate enhancement, and obstruction by encasement rather than mural infiltration favors lymphoma, especially with vessel encasement without occlusion, multistation lymphadenopathy or splenomegaly, and a known systemic lymphoma context

Lymphoma may involve the liver, bile ducts, or present as nodal disease causing biliary obstruction through extrinsic compression [5, 6]. Lesions are typically homogeneous, with mild enhancement and marked diffusion restriction (Fig. 13) [47]. Vascular encasement without true invasion and smooth ductal narrowing favor benignity, while CCA typically shows true vascular invasion and occlusion [5, 6]. Bulky lymphadenopathy and systemic disease favor lymphoma over CCA. PET/CT usually shows hypermetabolic involvement, particularly in aggressive subtypes, although indolent forms (e.g., MALT lymphoma) may demonstrate low uptake [5]. Histologic confirmation is required [6].

#### Metastatic nodal disease (extrinsic compression)

**Fig. 14:**
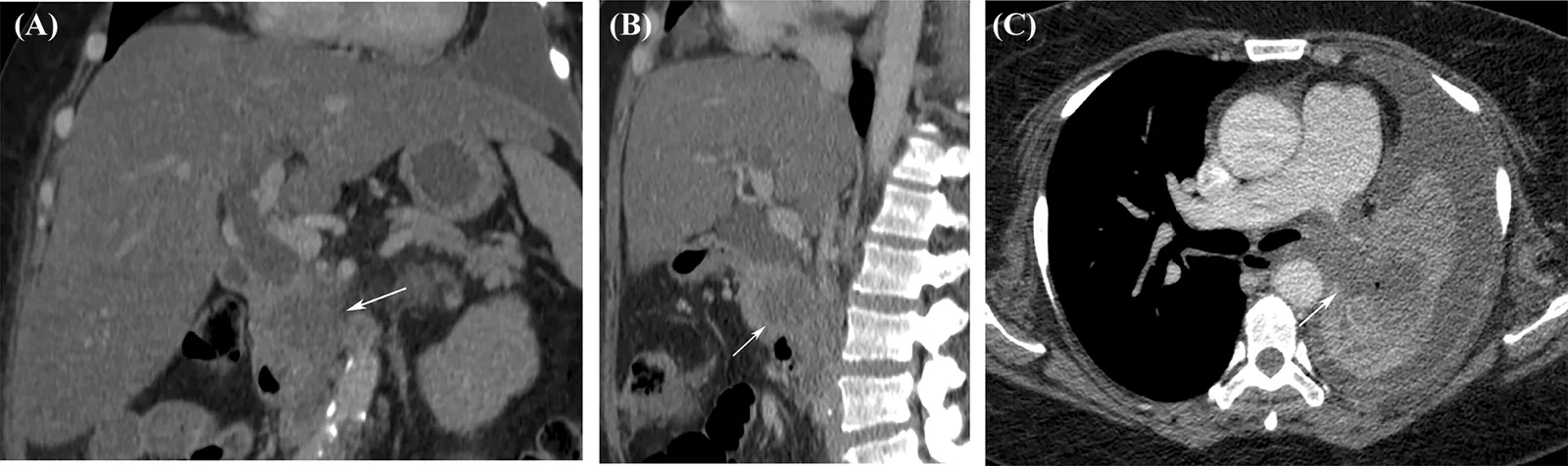
Metastatic nodal disease (extrinsic compression). **A**, **B** Coronal and sagittal portal venous phase CT reconstruction demonstrates heterogeneous lymphadenopathy compressing the distal portion of the common bile duct (arrow) with upstream biliary dilatation. **C** Chest CT image shows the primary pulmonary hilar mass, characterized by a heterogeneous lesion involving the left pulmonary hilum, associated with left pulmonary artery involvement, left lung atelectasis, and left pleural effusion (arrow). *Key features*: smooth extrinsic biliary compression by bulky, often necrotic conglomerate lymphadenopathy that displaces rather than infiltrates the duct, without a primary pancreatobiliary lesion, in a patient with a known non-HPB primary (colorectal, gastric, breast, lung, melanoma), favors metastatic nodal disease over cholangiocarcinoma or pancreatic adenocarcinoma. Histopathologic confirmation is required before treatment planning

Metastases from non-hepatopancreatobiliary primaries may cause biliary obstruction through peri-hilar masses, nodal compression, direct pancreatic or biliary metastasis, or peritoneal carcinomatosis [48]. Imaging typically shows hypovascular or heterogeneous lesions with ductal displacement or smooth compression rather than infiltrative stenosis (Fig. 14) [5, 6]. Multiple liver lesions, bulky lymphadenopathy, and known primary malignancy favor metastatic disease [6, 48]. Differentiation from CCA may require histologic confirmation, as imaging overlap can be substantial [48].

### Post-surgical and gallbladder related processes

#### Iatrogenic stricture

**Fig. 15:**
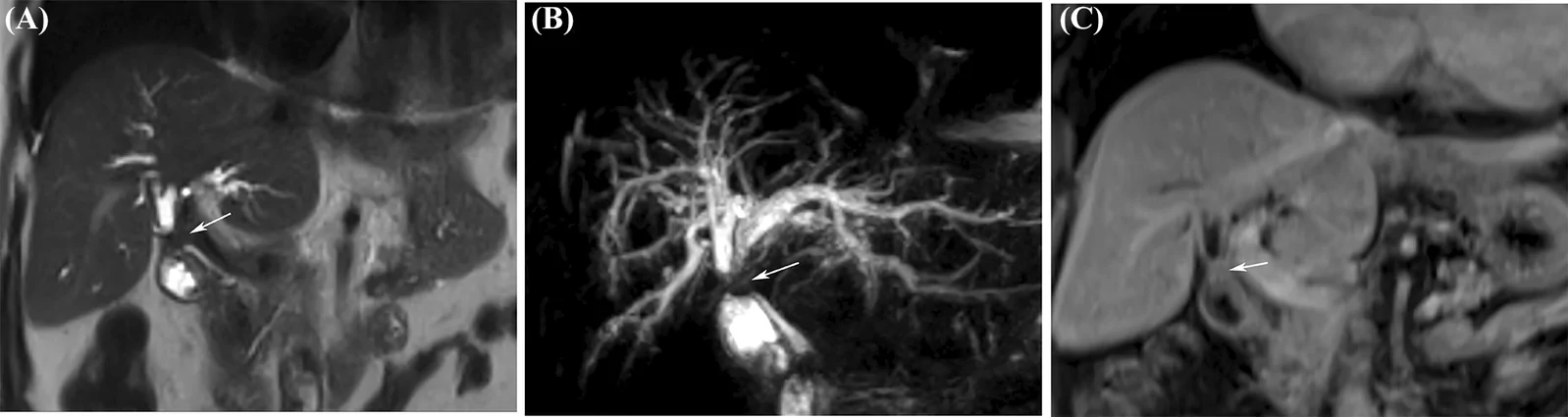
Iatrogenic stricture. **A** Coronal T2-weighted image demonstrates soft tissue thickening involving the mid-to-upper portion of the common bile duct (arrow) with distal narrowing and adjacent adhesions to the second portion of the duodenum. **B** MR cholangiography shows a stricture of the common bile duct (arrow) with upstream biliary dilatation. **C** Post-contrast coronal MR image demonstrates enhancement of the periductal soft tissue (arrow). Key features: focal, short-segment, smoothly tapered narrowing at surgical clips or a known anastomosis, most commonly post-cholecystectomy without a soft-tissue mass or progressive enhancement, strongly favors an iatrogenic etiology. Correlation with operative history, prior imaging, and temporal evolution is essential

Post-surgical and traumatic biliary strictures may present with focal ductal narrowing and upstream biliary dilatation, closely mimicking CCA. Neuromas are a rare late complication that may develop years to decades after cholecystectomy [5, 49]. On MRI and MRCP, these entities typically demonstrate smooth, segmental stenosis with relatively homogeneous mural thickening and mild enhancement (Fig. 15), in contrast to the irregular, asymmetric thickening and progressive enhancement more characteristic of CCA [5, 6]. However, imaging overlap is common, particularly in fibrotic strictures, with diagnostic accuracy of cross-sectional imaging in the range of 60–80% for biliary strictures. Correlation with surgical history is essential and, in indeterminate cases, histopathologic confirmation is necessary [39]. Specifically, correlation should document the type of procedure, time elapsed since surgery, and any biliary instrumentation. In indeterminate cases where imaging overlap with malignancy persists, tissue diagnosis through cholangioscopy or EUS-guided sampling should be pursued [39].

#### Xanthogranulomatous cholecystitis (XGC)

**Fig. 16:**
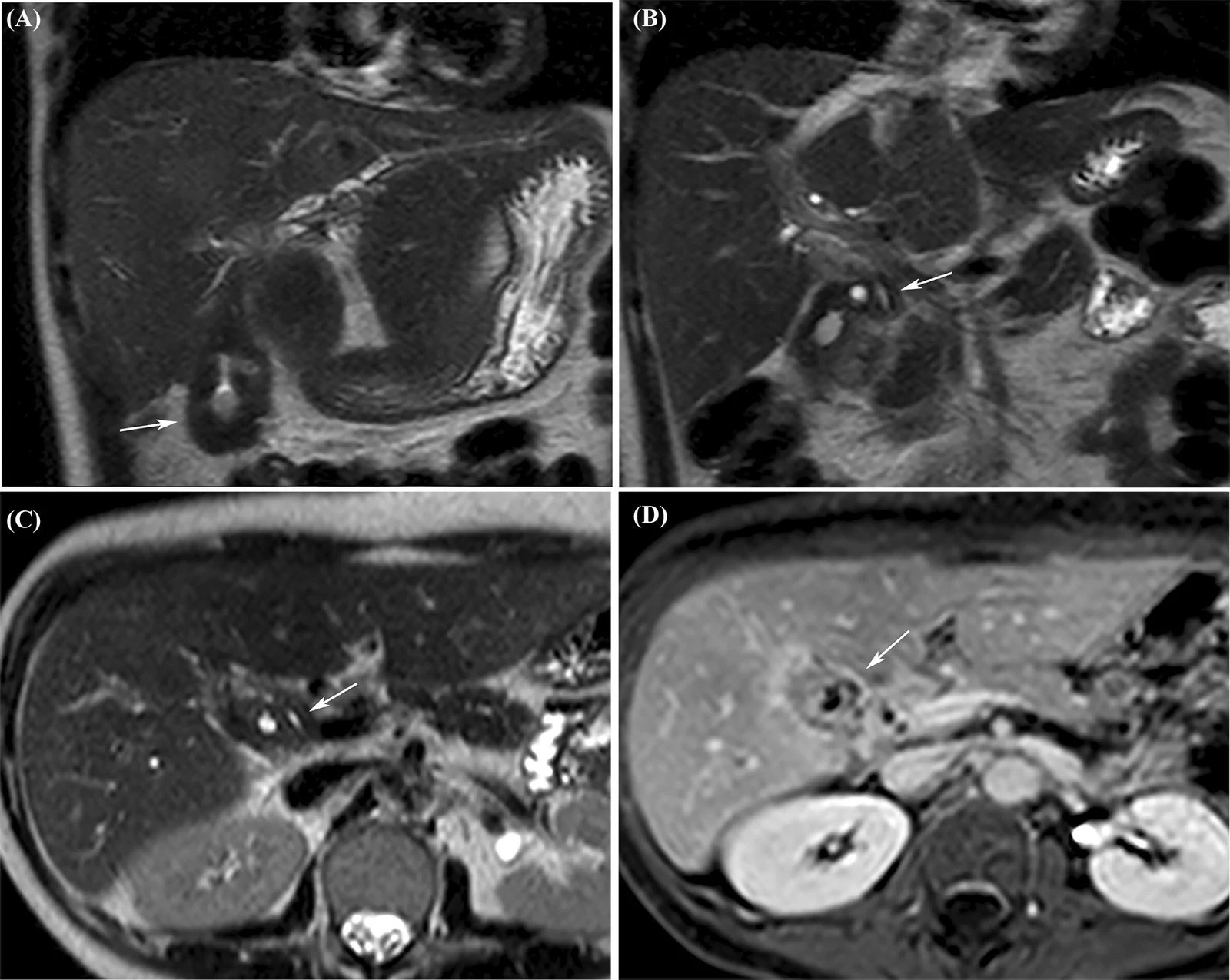
Xanthogranulomatous cholecystitis (XGC). **A** Coronal T2-weighted image demonstrates marked gallbladder wall thickening with predominantly hypointense signal intensity (arrow), suggestive of chronic inflammation. **B** Coronal T2-weighted image shows soft tissue thickening centered at the gallbladder infundibulum, extending toward the hepatic hilum and involving the mid portion of the common bile duct (arrow). **C** Axial T2-weighted image demonstrates the infiltrative soft tissue adjacent to the gallbladder infundibulum and surrounding the mid common bile duct (arrow). **D** Axial post-contrast portal venous phase T1-weighted image shows enhancement of the inflammatory soft tissue (arrow). *Key features*: diffuse gallbladder thickening with intramural T2-hyperintense, non-enhancing nodules, an intact mucosal line, contiguous inflammation respecting tissue planes, and cholelithiasis favors XGC over gallbladder carcinoma and cholangiocarcinoma

Xanthogranulomatous cholecystitis is an aggressive inflammatory condition that may extend to adjacent biliary structures and hepatic hilum, simulating malignant infiltration [5, 6]. Imaging typically shows diffuse gallbladder wall thickening with intramural hypoattenuating nodules and surrounding inflammatory changes; biliary obstruction may result from extrinsic compression or direct hilar extension (Fig. 16) [50, 51]. Features favoring XGC include diffuse wall involvement, intramural hypoattenuating nodules, preserved mucosal line, and associated cholelithiasis, whereas gallbladder carcinoma more commonly presents with focal wall thickening, an intraluminal mass, and lymphadenopathy [52]. When XGC extends to the biliary tree, differentiation from CCA may be particularly challenging, and histologic confirmation is often required [5, 50].

### Congenital: choledochal cysts

**Fig. 17:**
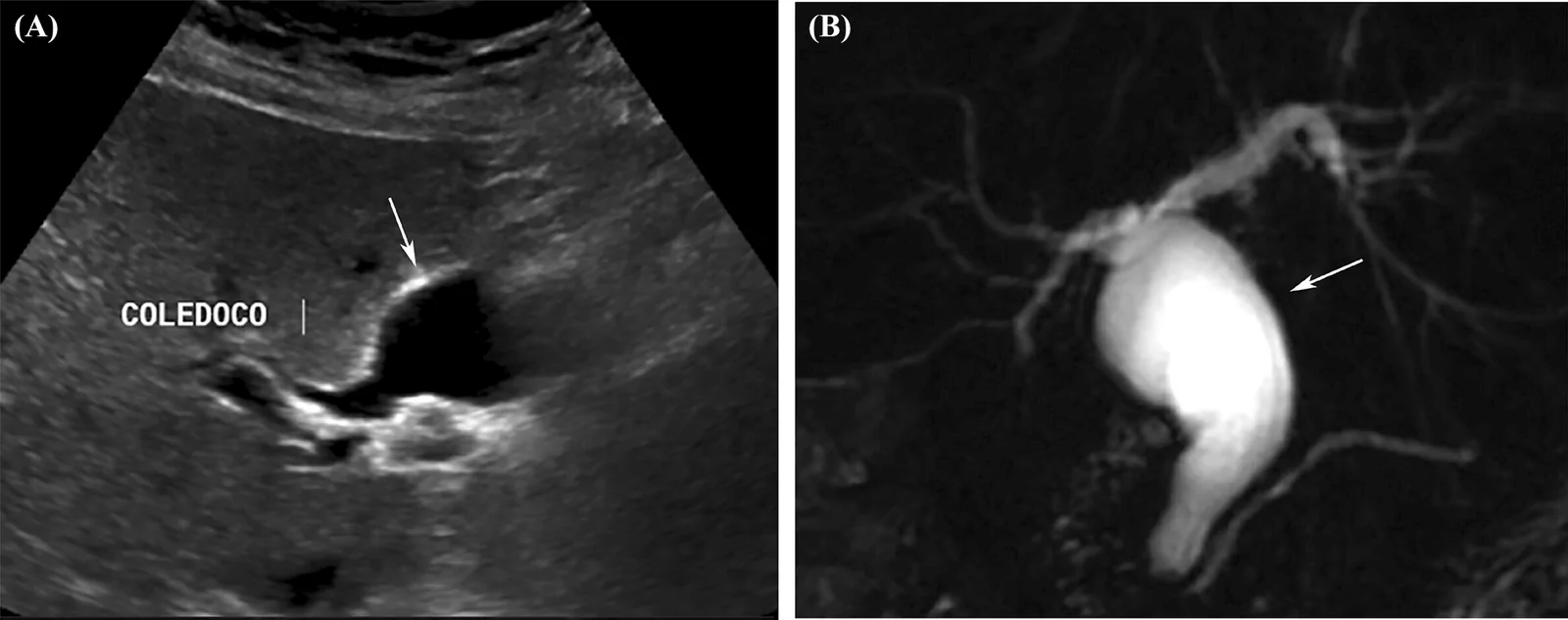
Choledochal cysts. **A** Ultrasound image demonstrates marked dilatation of the common bile duct without (arrow) significant intrahepatic biliary dilatation. **B** MR cholangiography shows fusiform dilatation of the common bile duct, predominantly involving its mid-to-upper portion (arrow), without significant upstream biliary dilatation or identifiable obstructive cause. *Key features*: fusiform/saccular extrahepatic cystic dilatation with smooth uniform walls, homogeneous T2-hyperintense contents without diffusion restriction or mural nodularity, plus an anomalous pancreaticobiliary junction, establishes the diagnosis and excludes intraductal-growing CCA/IPNB (which show mural irregularity, enhancing nodules with diffusion restriction, and heterogeneous mucinous contents)

Choledochal cysts present as fusiform or saccular biliary dilatation with smooth margins and gradual tapering, classified according to the Todani system based on anatomic extent [53]. On MRI, cyst contents are homogeneously T2 hyperintense without diffusion restriction, and the wall is typically thin with minimal enhancement (Fig. 17) [5, 6]. MRCP may also detect an anomalous pancreaticobiliary junction (APBJ), which strongly favors a congenital etiology [53, 54]. Features suggestive of CCA include abrupt ductal cutoff, irregular stenosis, asymmetric wall thickening, and progressive delayed enhancement. Periductal infiltration, vascular encasement, or lymphadenopathy further support malignancy [5, 6]. Importantly, choledochal cysts carry a significant risk of malignant transformation, between 7.5 and 30%, particularly types I and IV, with CCA being the most common malignancy, nearly 70%. New mural nodularity, wall irregularity, or enhancing soft tissue within a known cyst should raise suspicion for superimposed malignancy [53].

### Vascular compression and portal cavernoma-related disease

**Fig. 18:**
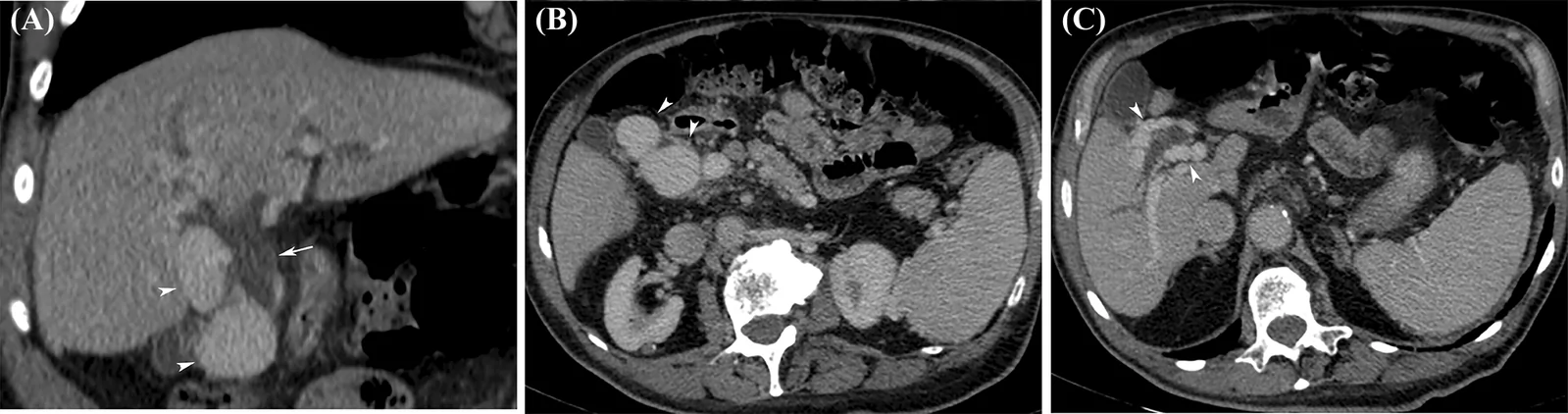
Portal cavernoma-related disease. **A** Coronal portal venous phase CT reconstruction demonstrates large hilar varices (arrowheads) compressing the mid portion of the common bile duct with upstream biliary dilatation (arrow). **B** Axial portal venous phase CT image shows large peribiliary varices (arrowheads) causing compression of the mid common bile duct. **C** Axial portal venous phase CT image demonstrates proximal common bile duct dilatation surrounded by multiple varices (arrowheads). *Key features*: serpiginous enhancing periductal and pericholecystic collateral vessels in the setting of cavernomatous transformation of an occluded portal vein, without mural thickening or a discrete soft-tissue mass, establishes the diagnosis. Characteristic MRCP findings include a “wavy” or “scalloped” duct contour, smooth shouldered narrowing, and variceal impressions mimicking stones

**Fig. 19:**
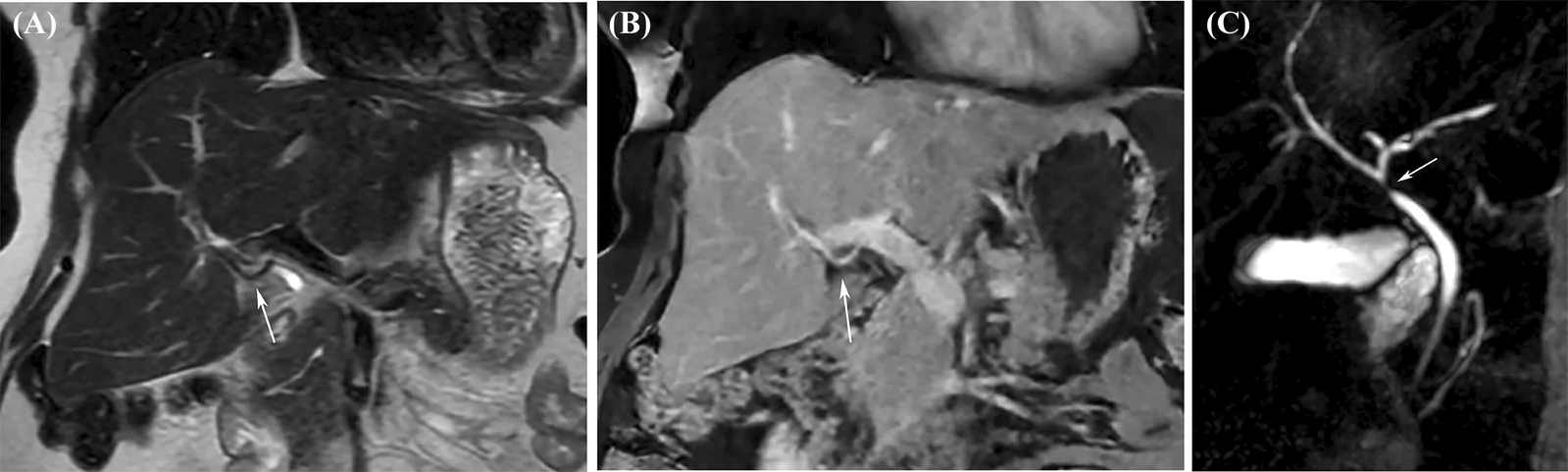
Vascular compression. **A** Coronal T2-weighted image demonstrates the proper hepatic artery coursing adjacent to the upper portion of the common bile duct (arrow). **B** Post-contrast coronal MR image confirms the vascular structure compressing the upper common bile duct (arrow). **C** MR cholangiography demonstrates focal signal loss/narrowing at the upper portion of the common hepatic duct (arrow) related to extrinsic compression by the proper hepatic artery, as identified in A and B. Key features: a focal, smooth, transverse extrinsic indentation of the CHD without upstream dilatation, filling defect, mural thickening, or periductal soft tissue, with direct anatomic correspondence to a crossing vessel (right hepatic artery most commonly) on contrast-enhanced sequences is diagnostic of vascular compression. The finding is incidental and clinically silent in the great majority of patients

Portal cavernoma cholangiopathy results from extrinsic compression of the bile ducts by periductal venous collaterals in the setting of chronic portal vein thrombosis, producing focal narrowing and upstream biliary dilatation that closely mimics CCA, a pattern historically termed the “pseudo-cholangiocarcinoma sign” [5, 6, 55]. On CT and MRI, serpiginous periductal collateral vessels appear as enhancing soft-tissue–like structures, sometimes forming a mass-like configuration (Fig. 18) [55]. MRCP typically shows segmental ductal narrowing with angulation, scalloping, and extrinsic indentation [56]. Narrowing due to extrinsic vascular compression, without mural irregularity or desmoplastic enhancement, favors a non-infiltrative process [5, 6]. Recognition of the periductal venous collaterals on contrast-enhanced imaging is key to diagnosis; MRCP coupled with MR portography provides simultaneous visualization of bile ducts and their relationship to the cavernoma [55] (Fig. 19).

## Conclusion

CCA typically presents with irregular, asymmetric ductal wall thickening and progressive enhancement reflecting its fibrotic stroma, frequently associated with abrupt upstream biliary dilatation and, in advanced cases, vascular encasement. Benign CBD strictures are usually short, smooth, and symmetric, with minimal or absent enhancement, preserved vascular relationships, and potential interval improvement following biliary decompression or anti-inflammatory therapy.

MRI with MRCP provides comprehensive ductal mapping and accurate mural characterization, while contrast-enhanced CT complements the evaluation of extrinsic compression, vascular involvement, and overall disease extent. An integrated interpretation of stricture morphology, enhancement pattern, upstream dilatation, periductal tissue involvement, and temporal evolution is essential for accurate differentiation.

In practice, a structured imaging approach improves diagnostic confidence. Malignant strictures are more often long, irregular, and enhancing, with abrupt upstream dilatation and associated with periductal soft tissue or vascular involvement. Benign entities typically demonstrate smooth tapering, minimal mural changes, identifiable underlying causes, such as calculi or inflammatory processes, and stability or regression over time.

Not every CBD dilatation, intraductal filling defect, or stricturing lesion represents CCA. A disciplined imaging framework, integrating MRCP anatomy with multiphase enhancement, diffusion-weighted imaging, evaluation of vascular relationships, systematic search for an underlying cause, and longitudinal assessment, is essential for distinguishing malignant from benign entities and for guiding optimal patient management.

Ultimately, when imaging findings remain indeterminate after comprehensive MRI/MRCP and CT evaluation, multidisciplinary discussion involving hepatobiliary surgery, gastroenterology, and radiology is strongly recommended. Tissue acquisition through ERCP with cholangioscopy-directed biopsy, EUS-guided fine-needle aspiration or biopsy, or intraductal ultrasound provides histopathologic confirmation and remains the definitive arbiter in diagnostically challenging cases. Radiologists play a central role in triaging patients toward appropriate invasive procedures by identifying which features are most suspicious and which mimickers must first be excluded.

## Data Availability

No datasets were generated or analysed during the current study.
